# Factors Influencing Farmer Willingness to Reduce Aggression between Pigs

**DOI:** 10.3390/ani9010006

**Published:** 2018-12-22

**Authors:** Rachel S. E. Peden, Faical Akaichi, Irene Camerlink, Laura A. Boyle, Simon P. Turner

**Affiliations:** 1 Animal Behaviour & Welfare, Animal and Veterinary Sciences Research Group, Scotland’s Rural College (SRUC), West Mains Rd., Edinburgh EH9 3JG, UK; Simon.Turner@SRUC.ac.uk; 2 Land Economy Environment and Society Research Group, Scotland’s Rural College (SRUC), West Mains Rd., Edinburgh EH9 3JG, UK; Faical.Akaichi@SRUC.ac.uk; 3 Institute for Animal Husbandry and Animal Welfare, University of Veterinary Medicine, Veterinärplatz 1, 1210 Vienna, Austria; Irene.Camerlink@vetmeduni.ac.at; 4 Teagasc, Pig Development Department, Animal & Grassland Research and Innovation Centre, Moorepark, P61 C997 Fermoy Co. Cork., Ireland; Laura.Boyle@teagasc.ie

**Keywords:** animal welfare, decision-making, farmers, pigs, structural equation model

## Abstract

**Simple Summary:**

Aggression between pigs is an important animal welfare issue in commercial farming, and is caused by an unstable social structure due to regular regrouping of unfamiliar pigs. The behavior has been extensively researched and several strategies to reduce aggression have been identified, however, they are not commonly used by farmers in practice. We conducted a survey of 122 UK and Irish pig farmers with the aim of understanding why farmers do not adequately implement aggression control strategies. This was important in order to identify targets for encouraging a change in practice. We found that the majority of farmers mixed pigs at least once during production and had tried at least one mitigation strategy in the past. However, farmers expressed limited willingness to implement strategies in the future, and this was influenced by: (1) their beliefs about the outcome of controlling aggression; (2) their perception of their ability to implement the necessary changes; (3) their perceptions of aggression as a problem and; (4) their views of relevant stakeholder groups. Based on these findings we make important recommendations on how to bridge the gap between research and practice.

**Abstract:**

Aggression between pigs remains an important animal welfare issue despite several solutions existing. Uptake of livestock welfare research relies on various stakeholders being willing to recommend or adopt changes to farm structure or management (e.g., veterinarians, researchers, farmers). This survey provides insight into the attitudes and practices of 122 UK and Irish pig farmers regarding aggression between growing pigs. Our aim was to understand why mitigation strategies are not adequately implemented. The majority of farmers mixed pigs at least once during production and had tried at least one mitigation strategy in the past. Farmers expressed limited willingness to implement strategies in the future, and a structural equation model revealed that this was directly influenced by their beliefs about the outcome of controlling aggression, and their perception of their ability to implement the necessary changes. Willingness was indirectly influenced by their perceptions of aggression as a problem and views of relevant stakeholder groups. Veterinarians had the greatest impact on farmer behavior. We recommend that researchers test research findings in practice, calculate cost-benefits of implementation, and transfer knowledge through various sources. This study showed that structural equation modeling is a valuable tool to understand farmer behavior regarding specific and entrenched animal welfare issues.

## 1. Introduction

Aggression between pigs is one of several animal welfare issues where there is resistance to change in practice despite extensive research [1,2]. The implementation of animal welfare research relies on a variety of relevant stakeholders. For example, researchers must effectively communicate their findings to the industry, and farmers and veterinarians must be willing to adopt these recommendations. It is therefore important to understand the decision-making process of farmers, in order to identify targets for initiating a change in practice. 

Pig aggression primarily occurs as a result of the unstable social structure created by regrouping, as pigs fight in order to re-establish dominance relationships [3]. Aggression threatens animal welfare and the economic sustainability of the production system through injuries such as skin lesions [3] and the transient effects of stress upon immune responses [4], growth rate [5] and susceptibility to infection [6]. For some producers, regrouping is unavoidable in order to maximize use of space. Furthermore, farmers attempt to create groups of pigs with equal body size which market simultaneously. Nevertheless, it is possible to limit the occurrence and intensity of aggression by implementing various mitigation strategies (review articles: [2,7,8,9]). For example, allowing litters to co-mingle (i.e., socialization) prior to weaning [10,11], housing pigs in relatively large groups [12], providing a diet high in tryptophan [13] and providing sufficient space [14] can all help control aggression at regrouping, but are employed by only a minority of farmers [2,15]. It is well established that a range of interrelated internal and external factors influence farmer behavior regarding animal welfare, such as personal values and attitudes, and financial and practical constraints (e.g., [16]). However, the literature on farmer behavior towards most welfare issues, including aggression, is scarce. This study aimed to: (1) explore the attitudes and behavior of UK and Irish pig farmers regarding aggression between pigs and; (2) investigate the factors that influence farmer willingness to implement aggression control strategies. The causal relationships amongst observed and latent variables were estimated by using structural equation modeling. We hypothesized that a range of interrelated factors influence farmer willingness to implement aggression control strategies. 

## 2. Research Hypotheses

We reviewed the literature on farmers’ decisions regarding animal welfare in order to formulate hypotheses on the factors that influence farmer willingness to control pig aggression. Much of this literature uses the framework of the Theory of Planned Behavior which describes willingness to make a behavioral change as being influenced by attitudes, perceived behavioral control and subjective norms [17,18]. The literature identifies wide ranging factors which can influence farmers’ animal welfare decisions, and the following hypotheses are representative of these findings.

**Hypothesis** **1.** *Perceiving positive outcomes of controlling aggression has a positive influence on farmer willingness to control pig aggression.*

A number of studies indicate that farmer decisions are influenced by beliefs about the outcome of an intervention, and the value the farmer gives this outcome [19,20,21]. Beliefs about the economic outcomes are particularly important; as the main incentive to participate in animal welfare schemes is an expected improvement in profitability and market access [21]. The most important barrier is distrust in the economic advantages of doing so [21,22].

**Hypothesis** **2.** *Perceiving the possibility to control aggression has a positive influence on farmer willingness to control pig aggression.*

Beliefs about the presence of factors that may facilitate or impede implementation of an animal welfare innovation are fundamental in farmers’ decision-making processes [19,20,23]. This relates to internal factors, such as a person’s perception of their own knowledge, skills and abilities, as well as external factors, such as time available and dependence on others. Farmer willingness to change current farm practice is limited when they perceive a lack of time, skilled labor, or knowledge [23,24,25], or if the welfare innovation is perceived to be unaffordable or difficult to practically manage [26].

**Hypothesis** **3.** *Valuing the opinion of relevant stakeholders regarding aggression has a positive influence on farmer willingness to control pig aggression.*

Putting a high value on the opinion of peers, researchers or specialists and feeling that peer-pressure and/or authorities expect improvements in animal welfare, is linked to greater willingness to provide high welfare standards [19,20,27]. Stakeholders differ in their impact on farmer behavior, with veterinarians being particularly influential [27] whilst researchers are distrusted [28].

**Hypothesis** **4.** *Perceiving aggression as a problem on their farm has a positive influence on farmer willingness to control pig aggression.*

Pig farmer perceptions are strong predictors of their behavior [29,30,31,32] and a recent survey of 167 UK pig farmers found that the majority do not perceive aggression as a problem that needs to be addressed [33]. As farmers are unlikely to invest resources in a change that they do not believe is necessary, this is likely to severely limit their motivation to control the issue. 

The literature directed us to these four specific hypotheses, however, since relatively little is known about the complex decision process we acknowledged that other causal links may exist that were not specifically hypothesized. Causal relationships among variables are likely to be complex and interrelated, for example, variables may influence farmer willingness to improve animal welfare directly or indirectly (through their effect on other variables, which subsequently directly influence willingness to improve animal welfare), and we take this into consideration during our statistical analysis. 

## 3. Materials and Methods

### 3.1. Data Collection

A total of 133 commercial pig farmers either completed or partially completed a survey (described in Section 3.2. Measures). Farmers were recruited by a range of means between February–December 2017. Firstly, a paper version of the survey was distributed via post to a sample of farmers that previously participated in aggression research conducted at Scotland’s Rural College (SRUC) (*n* = 170) and resulted in a 28.8% response rate (*n* = 49). Participants were also recruited at six farmer events organized by SRUC (*n* = 16), Teagasc (*n* = 28) and the Agricultural and Horticultural Development Board Pork (AHDB Pork) (*n* = 33). Finally, an online version of the questionnaire was created using Survey Monkey and the URL was distributed with the postal survey, and by contacts in the Pig Veterinary Society and the Weekly Tribune. All farmers were unaware of the project and unaware that they would be asked to complete the survey prior to being contacted. We chose to use both a paper and online survey in order to maximize our response rate by allowing farmers to choose their preferred method of participation. Responses to the online survey were low (*n* = 7). This is consistent with previous research which found a poor response of farmers to an online survey compared to a paper survey [34] and should be considered in future surveys of farmers. Those recruited at farmer events were unaware that they would be asked about the topic of aggression prior to attending, therefore these farmers were unlikely to introduce bias into the sample. However, those who voluntarily responded via post or online were likely to be progressive and interested in the survey topic. These farmers may, therefore, have introduced some bias into the sample.

### 3.2. Measures 

The survey comprised of three main sections entitled: (1) mixing aggression; (2) farm demographics; and (3) personal demographics (see Supplementary Materials). The first section contained all items used to measure each observed and latent variable included in the structural equation model (described in Section 3.4. Data analysis). These were three items measuring farmer outcome beliefs about controlling aggression, nine items to measure social influence, four items to measure perceived possibility to control aggression, one item to measure participants’ perception of aggression as a problem on their farm and one item to measure their willingness to implement aggression control strategies. They were also asked which aggression control strategies they had tried and how useful they found them to be (from 1 = ‘not useful at all’ to 7 = ‘very useful’) (all statements used in the model are shown in Table 1). In addition, farmers were asked for their level of agreement/disagreement (from 1 = ‘strongly disagree’ to 7 = ‘strongly agree’) with the statement ‘mixing aggression is a problem for the industry’. By using a 7-point Likert type scale, farmers were not forced to agree/disagree as they were able to provide a neutral response (4 = ‘neutral’). We chose to include the neutral response because farmers may have been either unopinionated or unfamiliar with certain content. Farm information was collected regarding location, quality assurance scheme membership, farm size (number of pigs), housing system (indoor/outdoor/combined), average group size, and at what stages of production pigs are mixed. Personal demographic information was collected regarding age, gender, role on farm, and years of experience working with pigs. Prior research shows that farmers interpret tail biting and mixing aggression as interchangeable terms [28,33] even though the two behaviors have different causes [35,36]. Therefore a clear definition of mixing aggression was provided, and participants were instructed not to include tail biting in their answers. The survey was piloted with 8 pig farmers and 17 researchers and amended according to their feedback regarding the wording and appropriateness of questions. This study was conducted in accordance with the Declaration of Helsinki. This study received internal ethical approval from the Human Ethical Review Committee at the University of Edinburgh (Project identification code: HERC_66_16), and informed consent was obtained for all participants. 

### 3.3. Socioeconomic Characteristics of the Final Sample

It was necessary for the full dataset to be analyzed and therefore participants who only partially completed Section 1 of the survey were removed. Of the 133 responses, only 122 completed all questions, and the others were not included in the data analysis. The majority of the final respondents were male (73.8% male; 12.3% female; 13.9% undisclosed), on average 47 ± 15.05 years old (min 17; max 82) and with 26 ± 16.24 years of experience working with pigs (min 1; max 70). Of the respondents, 41.8% were farm owners, 18.9% were managers and 23% were farm workers. The remaining respondents were contract farmers (4.1%), retired (5.7%) or chose not to disclose their role (6.6%). All farmers, regardless of their role on the farm, were capable of making changes to pig husbandry which would directly influence aggression (e.g., by avoiding mixing unfamiliar pigs). Opportunities to make changes may be more constrained for those who do not have a management role. Nevertheless, the responses of all of the surveyed farmers were relevant due to their current or past experience of pig husbandry. 

The study was carried out across the UK and Ireland, with 54.1% of respondents based in England, 16.4% in Scotland, 2.5% in Wales, 3.3% in Northern Ireland, 18.9% in the Republic of Ireland and 4.8% undisclosed. Red Tractor was the most common quality assurance scheme, assuring 54.1% of the surveyed farms, followed by RSPCA (23.8%), Assured British Pigs (14.8%), Quality Meat Scotland (13.9%), Scottish SPCA (8.2%) and Genesis Quality (6.6%). Some of the farmers reported having no quality assurance scheme (13.9%), whilst 11.5% indicated that they were assured by another scheme (ticked ‘other’) but they did not specify which one. Some farms were assured by more than one scheme.

Farms on average kept 3625 growing/finishing pigs at any given time, and the majority of pigs were housed indoors (see Table 2 for information on farm size and housing system). Of the 71 farmers who reported average group size for growing/finishing pigs, only 25 kept pigs in conventional groups of 25 or smaller. On average growing/finishing pigs were kept in groups of 85.6 ± 121.92 (min 12; max 600). Of the 122 participants, 74.6% were farrow-finish, 3.3% were wean-finish and 4.9% were grow-finish; whilst 16.4% did not provide sufficient information.

The sample is roughly representative of UK agricultural workers which are mainly males around 59 years of age [37]. Farms showed a wide range in size and were based mainly in England, which is also representative of the UK population (53% of the UK breeding farms have between 250 and 749 sows and 85% are based in England [38,39]. 

### 3.4. Data Analysis

#### 3.4.1. Overview

The method used in this study was Structural Equation Modeling (SEM) which has been used previously to explore the attitudes and behavior of farmers [19] and consumers [40] towards animal welfare in general. The method has also been used to explore farmer decisions regarding specific issues such as nitrate pollution [41] and participation in organic farming programs [42]. However, this method has never been used to tackle farmer behavior regarding specific and entrenched animal welfare issues. SEM uses observed and latent variables whereby the latent variables are not directly observed but are inferred from multiple indicator variables. Observed variables are single indicator variables. SEM tests the causal links specified in a theoretical model and describes the nature and magnitude of the relationship between latent and observed variables [43]. SEM takes into account both direct and indirect causal relations. To test the proposed model the two-step approach proposed by [44] was used. Firstly, confirmatory factor analysis was employed to confirm that observed variables (i.e., responses to specific items) loaded onto latent variables in a manner that supported the definition of the latent variable (e.g., outcome beliefs about controlling aggression). This step defined the measurement model. The second step involved assigning the relevant relationships among variables (latent and observed) to build and test the structural model. Therefore the term SEM refers to the full model which consists of two types of model: the measurement model and the structural model. These steps are described in more detail in following sections. SEM analysis was conducted using the statistical package Stata (version SE 15, StataCorp, TX, USA). 

All additional analyses were conducted using SPSS (version 22, International Business Machines Corp, Armonk, NY, USA). Specifically, associations between items on a 7-point Likert type scale were assessed using Spearman Rank correlation coefficient. Wilcoxon signed-rank tests were used to determine the median difference between two paired or matched observations (e.g., farmer agreement with the statement ‘mixing aggression is a problem on my farm’ compared to ‘mixing aggression is a problem for the industry’). Friedman tests were used to determine median differences between three or more matched observations (e.g., comparing farmer responses to ‘How much does the opinion of this person/group affect your decisions with regard to mixing aggression?’ for each of the nine stakeholder groups). Kruskal-Wallis tests were used to determine differences between three or more independent observations (e.g., comparing the perceived usefulness of each of the nine aggression control strategies as judged by the specific farmers who had tried them). Significant results were followed up with pairwise comparisons with a Bonferroni correction for multiple comparisons. To determine the impact of farm location (England, Scotland, Wales, Northern Ireland, Republic of Ireland) on practice regarding aggression we ran a chi-square test whereby the dependent variable was experience of trying an aggression control strategy in the past (yes/no). To determine the impact of farm location on willingness to implement aggression control strategies we used cumulative odds ordinal logistic regression with proportional odds. The assumptions of (i) no collinearity between independent variables and (ii) proportional odds were checked through inspection of collinearity diagnostics and the full likelihood ratio test. To determine whether there was a relationship between farm size (number of growing/finishing pigs) and willingness to implement aggression control strategies we used Spearman’s rank correlation coefficient. Results were considered significant where *p* < 0.05 for all tests. Where responses on the 7-point Likert type scale were pooled (e.g., in order to describe the percentage of participants to agree with a statement); points 1–3 were coded as ‘disagree’; 4 was coded as neutral and; 5–7 were coded as ‘agree’. 

#### 3.4.2. Measurement Model (MM)

The first step involved performing confirmatory factor analysis for the following latent variables: (1) outcome beliefs about controlling aggression (OB); (2) social influence (SI) and; (3) perceived possibility to control aggression (PP). The purpose of this step was to confirm that variables assumed to describe a latent variable did so (see Table 1 for individual variables included in each factor). We undertook separate confirmatory factor analysis (CFA) for each latent variable. Convergent validity of each latent construct was verified by checking: (1) the magnitude and direction of standardized factor loadings which indicate the relationship of each variable to the underlying factor (a minimum threshold of 0.5 was set); (2) the Cronbach alpha coefficient which indicates internal consistency of variables within a factor (which should be >0.6); (3) the average variance extracted (AVE, which should be >50%) and; (4) composite reliability which provides another measure of internal consistency (CR, which should be >0.7) [45,46]. Discriminant validity indicates whether variables that are not supposed to be related are actually unrelated and was checked by assessing the squared correlation between latent constructs (SC, which should be <AVE). To check validity of the measurement model a range of diagnostic parameters were used. Firstly, the ratio of the chi-square (*χ*^2^) to the degrees of freedom (*df*) should not exceed 3. We also checked the root mean square error of approximation (RMSEA, which should be <0.05), the comparative fit index (CFI, which should be <0.90) and the standardized root mean squared residual (SRMR, which should be <0.08) [47]. 

#### 3.4.3. Structural Model

After obtaining a satisfactory measurement model, we defined and ran the structural model using robust maximum likelihood analysis. The purpose was to estimate the relationships among and between number of strategies tried (NST), perception of aggression as a problem (PERC), willingness to control aggression (WILL) and the latent variables (OB, SI and PP) in order to test our hypotheses and advance our understanding of the complex relations among constructs. The literature directed us to four specific hypotheses (Section 2), however, since relatively little is known about the complex decision process we acknowledged that other causal links may exist that were not specifically hypothesized. Therefore we did not constrain the SEM to look only at the relationships hypothesized a priori to exist, and we estimated all possible relationships between NST, PERC, WILL, OB, SI and PP. 

## 4. Results

### 4.1. Attitudes and Current Practice

**Figure 1:**
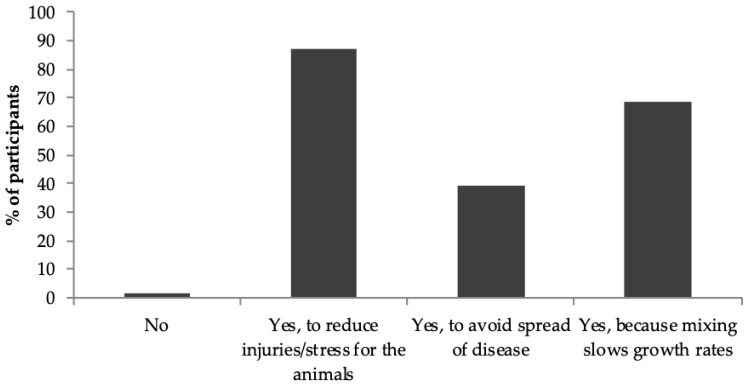
Percentage of farmers to tick each statement in response to the following question: ‘Do you avoid mixing unfamiliar growing/finishing pigs as far as practically possible? Please tick all that apply’.

Almost all of the respondents (99.2%) reported avoiding mixing growing/finishing pigs wherever possible (see Figure 1). Nevertheless, only 5.7% of farmers reported never mixing unfamiliar growing/finishing pigs during production; 45.9% of farmers reported mixing once, 28.7% mix twice, 6.6% mix three times and one farmer mixed four times during each production cycle. Growing/finishing pigs were most commonly mixed at weaning (56.6%), followed by grower (28.7%), slaughter (23.8%) and finisher stages (16.4%). Nine percent of the farmers also indicated that they regroup pigs at other stages of production. Of those that specified the timing of this mixing, both reported regrouping the remaining pigs that were not yet big enough to be sent for slaughter (*n* = 2). 

Farmers were more likely to agree that aggression was a problem for the industry (51.6% agreed; median response = 5) than for their own farm (14.8% agreed; median response = 3) (*z* = 5.582, *p* < 0.0001). Farmers (84.4%) were aware of methods to reduce mixing aggression. Half of them (50%) believed they had the possibility to make changes in management and 38.5% believed that they had time available to reduce mixing aggression. Of the farmers who indicated having responsibility for making financial decisions (*n* = 88), 39.8% believed that they had the financial resources to control mixing aggression. Most farmers (88.5%) had tried at least one strategy to control aggression when mixing growing/finishing pigs, with respondents selecting between one and nine techniques (1: *n* = 8; 2: *n* = 17; 3: *n* = 30; 4: *n* = 22; 5: *n* = 14; 6: *n* = 8; 7: *n* = 4; 8: *n* = 4; 9: *n* = 1). There was no effect of farm location (country) on likelihood of having tried a strategy (*p* > 0.05). The most used strategy was distraction material/toys (73.8%) whilst the least used strategy was the application of sedation (13.1%) (Table 3). There was a statistically significant effect of strategy on the perceived usefulness of each strategy (*χ*^2^ (8) = 21.016, *p* < 0.05). Pre-weaning socialization was perceived as more useful than mixed weight pens (*p* < 0.05) and purposely mixing from neighboring pens (*p* < 0.05) (Table 3). In addition to those strategies listed in the survey, farmers also used the comments section to emphasize the important link between genetics and aggression (*n* = 3). Moreover, two farmers reported using water sprinklers to control aggression at mixing.

**Figure 2:**
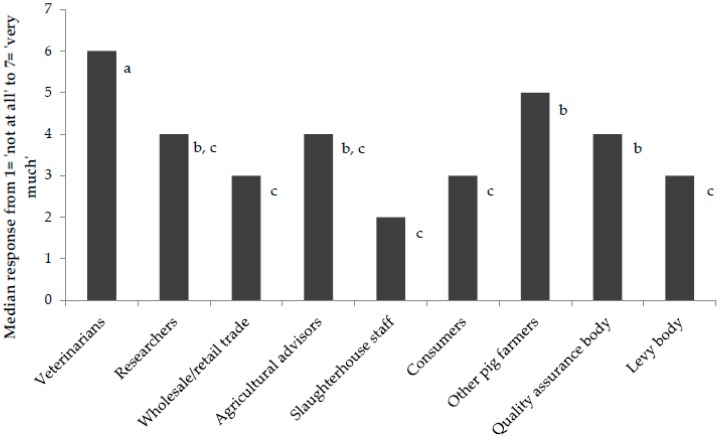
Farmers’ median response to the question: ‘How much does the opinion of this person/group affect your decisions with regard to mixing aggression?’ (from 1 = ‘not at all’, to 7 = ‘very much’). Letters indicate results of post-hoc pairwise comparisons whereby different letters indicate a significant difference between groups at *p* < 0.05.

More than half of the participants (54.9%; *n* = 67) believed that a reduction in mixing aggression would create more profit for their farm, whilst 58.2% (*n* = 71) believed it would improve animal welfare and 57.4% (*n* = 70) believed it would improve their job satisfaction. Farmers stated that they were influenced by different stakeholders to different degrees (*χ*^2^ (2) = 195.917, *p* < 0.001). Veterinarians had a greater influence on farmer behavior than any other stakeholder (*p* < 0.01), whilst other pig farmers and quality assurance bodies had a significantly greater influence than the wholesale/retail trade, slaughterhouse staff, consumers and levy bodies (*p* < 0.01) (see Figure 2). Fewer than half (41%) of participants indicated that they were willing to implement aggression mitigation strategies in the future. This was unaffected by farm location (*p* > 0.05) or number of growing/finishing pigs (r = −0.132, *p* > 0.05).

### 4.2. Measurement Model

As mentioned in Section 2 the first step of the SEM was to carry out confirmatory factor analysis to identify latent constructs for: (1) outcome beliefs about controlling aggression (OB); (2) social influence (SI) and; (3) perceived possibility to control aggression (PP). Confirmatory factor analysis revealed that the four PP questionnaire items did not load onto a common construct, and this could not be improved by adding or removing individual variables. As a result, we employed PP_2_ (‘I or my colleagues have the possibility to make changes in management which would reduce mixing aggression’) as an observed variable; of the four PP items PP_2_ was deemed the most capable of fully representing this construct. We identified and extracted the two remaining latent variables (SI and OB) measured by 12 indicator variables. Reliability of factor loadings were high (standardized factor loadings were above 0.5) for all observed variables forming these latent constructs with the exception of SI_2_ which fell below the 0.5 threshold at 0.41. The slightly lower standardized factor loading for this variable is due to the fact that veterinarians (SI_2_) had a significantly greater influence on farmer behavior when compared to any other stakeholders (Figure 2). This variable was retained in the model due to its theoretical significance as indicated by prior research [27]. The Cronbach alpha coefficient (>0.6), CR (>0.7) and AVE (>50%) all confirmed good internal consistency for both latent variables [45,46]. Results of the standardized factor loadings, average variance extracted (AVE) and composite reliability are shown in Table 4. Discriminant validity was confirmed by assessing the SC between latent constructs (which should be <AVE). Validity of the measurement model was confirmed through the *χ*^2^ and the ratio *χ*^2^/*df* (<3), RMSEA (<0.08), CFI (>0.9) and SRMR (<0.08) which all indicated a good model fit (see Table 4). 

### 4.3. Structural Model 

**Figure 3:**
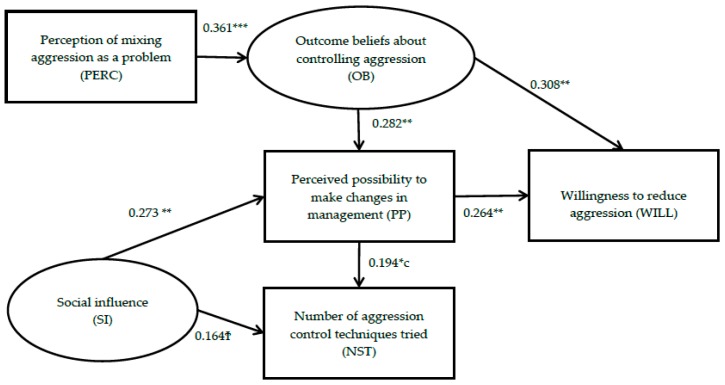
Path diagram of standardized parameters, whereby; ^Ϯ^ *p* < 0.1, * *p* < 0.05, ** *p* < 0.01, *** *p* < 0.001. Latent variables are represented by ovals and observed variables by rectangles. All possible relationships between observed and latent variables were estimated and only relationships whereby *p* < 0.10 are displayed. Regression coefficients are presented for each relationship.

After a satisfactory measurement model was obtained, a structural model was estimated. The results can be seen in Figure 3. Latent variables are represented by ovals and observed variables are represented by rectangles in the path diagram. All possible relationships between observed and latent variables were estimated, and only relationships whereby *p* < 0.10 are displayed. All regression coefficients presented were positive. The regression coefficient revealed that OB was the largest determinant of WILL; furthermore OB affected WILL indirectly through PP. Therefore H1 (perceiving positive outcomes of controlling aggression has a positive influence on farmer willingness to control aggression) is supported. The regression coefficient of PP on WILL was also positive and significant. Therefore H2 (perceived possibility to control aggression has a positive influence on farmer willingness to control aggression) is also supported. The direct regression coefficient of SI on WILL was not significant; therefore H3 (valuing the opinion of relevant stakeholders regarding aggression has a positive influence on farmer willingness to control aggression) is not entirely supported. However, there was a significant indirect relationship between SI and WILL through PP. The direct regression coefficient of PERC on WILL was not significant; therefore H4 (Perceiving aggression as a problem on their farm has a positive influence on farmer willingness to control pig aggression) is not entirely supported. However, there was a significant indirect influence of PERC on WILL through OB. In addition, results revealed a significant, positive relationship between PP and NST. PERC and OB indirectly influenced NST through PP and the regression coefficient of SI on NST was also positive and approaching significance (*p* = 0.07). Spearman correlation matrix among variables used in the structural model can be seen in Appendix A.

## 5. Discussion

Progress in uptake of advice from animal welfare research largely depends upon the willingness of farmers to adopt changes to farm structure or management. This survey of 122 pig farmers yields new insight into the attitudes and practices found in the UK and Irish pig industries regarding the longstanding animal welfare issue of aggression between pigs. Furthermore, we provide the first investigation into the factors that influence farmer willingness to control aggression. This new insight into farmer decision-making provides tools for bridging the gap between research and practice, which has so far been resistant to change [2]. 

### 5.1. Perceptions and Practice Regarding Aggression

All but one participant reported avoiding mixing unfamiliar growing pigs whenever possible. Despite this, the vast majority of farmers mixed growing pigs at least once during production, and for some up to four times. Farmers were aware that aggression is associated with welfare and production costs as the primary motivations for avoiding mixing were concerns over injury/stress for the animals and slowed growth rates. However, only 14.8% of participants perceived that aggression between unfamiliar growing/finishing pigs was a problem on their farm. This finding is consistent with a previous survey showing that the majority of UK pig farmers did not perceive aggression as a problem that needed to be addressed [33]. Nevertheless, 51.6% of surveyed farmers in the current study perceived that aggression is a problem for the industry. Therefore the majority of farmers believe that they sufficiently control aggression at mixing on their farm, and seem unwilling to acknowledge an issue with their own production system or are indeed successfully controlling aggression despite mixing pigs. 

Aggression mitigation strategies have been researched since the 1970s. They display mixed efficacy results in empirical trials, reviewed several times [2,7,9,48]. The majority of respondents were aware of aggression mitigation strategies and 88.5% had tried at least one strategy to control aggression at mixing in the past. Farmers found pre-weaning socialization to be more useful than employing mixed-weight pens and mixing pigs from neighboring pens, the latter two being the only strategies that were judged not useful. Farmers perceived all other aggression mitigation strategies as comparably useful. 

For some strategies, their perceived usefulness in the current study is consistent with their usefulness in the peer-reviewed literature. Mixing pigs from neighboring pens was proposed to reduce aggression by allowing pigs to become familiar with one another prior to mixing, but it’s efficacy for reducing aggression has received inconsistent support from a small number of empirical trials [7,49,50] and it was not judged useful in the current study. Pre-weaning socialization is one of the most promising strategies to control aggression identified by research [2] and was judged the most useful strategy in the current study. A recent experiment showed that pre-weaning socialization was beneficial also on a large scale commercial farm [11] and such information is a useful addition to the studies done under research conditions. However, despite its effectiveness, only 38.5% of surveyed farmers had tried it, although uptake was slightly higher than reported in a previous survey of 167 UK pig farmers whereby 27% of farmers with breeding sows employed the strategy [33]. The limited uptake of this strategy may be due to concerns about its practical management including increased work load, pathogen spread, growth and mortality [33]. The perceived usefulness of housing pigs in large social groups is also in agreement with the research, which suggests that pigs adopt a less aggressive social strategy when the costs associated with aggression outweigh the benefits [51,52]. Group size must be sufficiently large (more than 12 individuals) to have an impact on aggression levels [51] but much larger groups (>80 pigs) are more effective [12,52]. Average group size for growing/finishing pigs in the current study exceeded 80, illustrating the move towards larger group sizes observed in industry and driven by reduced cost, ease of management [53] and by the development and launch of automatic sorting technology which requires large groups [54]. 

The participants’ judgment that some strategies were somewhat useful is inconsistent with the research evidence. The most commonly used strategy was distraction using materials/toys, despite there being no evidence supporting its effectiveness. This could be linked to the fact it is relatively cheap and easy to add available materials to pens. Provision of enrichment is a requirement of EU Council Directive 2008/120/EC to control tail biting, but it must be used appropriately in order to avoid an increase in aggression caused by easily defensible resources, i.e., a sufficient number of objects should be present to ensure adequate access [55]. As farmers reported this strategy to be useful in controlling aggression, it is possible that they still considered tail biting as aggression. It may also be that research has largely ignored practical low-cost strategies that have emerged from practice, such as providing roughage at regrouping. Odor masking agents, which disrupt the olfactory processes by which pigs recognize each other, and barriers/get-away areas, which allow pigs to escape, show somewhat mixed results in research [2,7,56]. Moreover, tranquilizers [57] and evening/night-time mixing [58] appear to delay rather than reduce aggression [2,7]. Mixed-weight groups were not judged useful here although they do appear to reduce aggression in empirical trials by allowing dominance hierarchies to be clearly determined by physical size and strength [3]. This lack of correspondence between strategies found to be useful in research and in industry was also observed in a previous study [15] whereby the author suggested that other influences are involved, such as differences in the associated costs and ease of implementation influencing their relative perceived benefits. Indeed, mixed-weight pens restrict access to resources for low weight pigs resulting in increased divergence in weight as pigs grow [7,59]. Having pigs reach slaughter-weight at different times results in less efficient use of space and requires further regrouping of smaller pigs left behind. Therefore, their lack of usefulness could be associated with the fact they are not practical to manage in a commercial setting. 

Two farmers used shower sprays/water sprinklers to control aggression at mixing. This is encouraged in the code of recommendations which must be read and understood by all farmers in the UK [60,61,62] despite no peer reviewed research existing that has investigated the use of this strategy.

### 5.2. Factors Influencing Farmer Willingness to Implement Aggression Mitigation Strategies

Despite the fact that most farmers had tried at least one aggression mitigation strategy in the past, only 41% of participants indicated willingness to implement aggression control strategies in the near future. Farm location had no impact on farmers’ use of aggression control strategies in the past or their willingness to control aggression in the future; suggesting similar practice between Ireland and counties within the UK. SEM analysis confirmed that a range of interrelated internal and external factors influenced the number of aggression mitigation strategies tried in the past and farmer willingness to implement strategies in the future. Willingness to act in the future was directly influenced by beliefs about the consequences of controlling aggression and whether farmers felt able to implement the change (therefore supporting H1 and H2). The value of relevant stakeholder opinion, and perceiving aggression to be a problem on their farm, did not directly influence farmers’ willingness to implement aggression control strategies (therefore not supporting H3 and H4). However, both the value of relevant stakeholder opinion, and perceiving aggression to be a problem on their farm, did indirectly effect willingness through enhancing farmers’ perceived possibility to implement strategies and by enhancing beliefs about the beneficial outcomes of controlling aggression, respectively. 

Farmers’ own perceptions about their ability to make changes in management played a central role in their willingness to implement aggression control strategies. It was directly affected by both outcome beliefs and social influence and, in turn, had a direct effect on both willingness to implement aggression control strategies in the future and the number of strategies tried in the past. This is consistent with previous research whereby farmers’ decisions regarding animal welfare were influenced by their beliefs about the presence of factors that may facilitate or impede implementation of a particular change [19,20,23]. Implementation of aggression control strategies could therefore be stimulated by enabling farmers to make changes or by giving them the confidence that change is possible.

Beliefs about the outcomes of controlling aggression were the biggest determinant of farmer willingness to implement aggression mitigation strategies. One solution would therefore be to prove whether research results can be consistently replicated in practice and yield benefits that would outweigh potential costs. This can be achieved by testing strategies under commercial conditions and by conducting cost-benefit analyses. Outcome beliefs were directly limited by the fact that the majority of farmers did not perceive aggression to be a problem on their farm. Therefore, an important target for intervention is providing farmers with the necessary knowledge and tools to accurately recognize when aggression is an issue, for example by encouraging farmers to observe the number and location of lesions which can provide a quick and well established estimate of aggressive behavior and can help farmers to identify the extent of the problem on their farm [63,64]. Farmer beliefs regarding the financial outcomes of controlling aggression are likely to be limited further as the economic consequences of aggression have never been calculated. Uncertainty about price premiums is a major barrier to investment in animal welfare [22,65]; therefore research is required to establish the cost-effectiveness of aggression mitigation strategies in order to predict the outcome of interventions on farm profitability. The SEM analysis reveals that by targeting outcome beliefs, farmers may perceive the possibility to make changes in management as greater. 

Relevant stakeholder groups influenced farmers’ perceived possibility to make changes in management, highlighting another important target for implementing changes on-farm. Interestingly, stakeholders did not influence the likelihood that farmers would perceive aggression on their farm to be a problem, or their beliefs about the benefits of controlling aggression. This highlights that current methods of communicating with farmers on these specific issues requires improvement. Veterinarians had the greatest influence, which is consistent with the fact that veterinarians are the most obvious point of call for advice regarding harmful issues such as aggression, and they were previously identified by pig farmers as their most trusted and preferred source of information [27]. Researchers, other pig farmers, agricultural advisors and quality assurance bodies all had a lower, but potentially important, influence on farmer behavior regarding aggression. The wholesale/retail trade, consumers, levy bodies and slaughterhouse staff did not drive industry towards change on this matter and this is linked to the fact that pig aggression is either unknown to these groups or not a priority to the majority of these groups, who are known to lack engagement with specific animal welfare issues on the farm (e.g., retailers [66,67]; consumers [65,68,69]; slaughterhouse staff [70,71]). Therefore, researchers should effectively communicate their findings not only to farmers, but to a variety of stakeholder groups, with special emphasis on veterinarians. To be effective, researchers should tailor their communication based on the knowledge base of their audience and move away from the traditional, unidirectional lecture format [72,73].

Applying SEM provided insight into farmer decisions regarding pig aggression. We had a relatively small sample size for SEM analysis, but comparable to previous published research employing SEM (e.g., [74]; *n* = 101). Recruitment of pig farmers for surveys remains difficult [34] and studies must be carefully designed to avoid bias towards including only proactive farmers. Other factors may have impacted farmers’ decisions about animal welfare as well, such as their immediate family and personality characteristics [16]. The SEM approach was powerful for analyzing the direct and indirect links between a multitude of social factors and we recommend applying this method to other important animal welfare challenges. 

## 6. Conclusions

The majority of respondents were aware of aggression mitigation strategies and had tried at least one strategy to control aggression in the past. Strategies were generally thought of as moderately useful but the majority of farmers were reluctant to employ strategies in the near future. Structural equation modeling analysis showed that farmer willingness to make a change was directly influenced by their beliefs about the outcome and their perceived possibility to make a change. Furthermore, their perceptions of the problem and relevant stakeholder groups indirectly influenced willingness to make a change. We recommend that researchers employ a multipronged approach to encourage implementation of control strategies. By testing aggression control strategies outside of the highly controlled research setting it will be possible to establish their outcomes and practical implementation under commercial conditions. By calculating the economic consequences of aggression mitigation it will be possible to advise farmers on the most cost-effective solutions and their impacts on farm profitability. Finally, information on the consequences of aggression, how to recognize it as a problem and how to control the issue must be effectively transferred into industry by researchers. Knowledge transfer should be directed not only at farmers but should be channeled through various sources, with special emphasis on veterinarians. The SEM approach could also be successfully employed to understand other entrenched welfare challenges in livestock production where there is inadequate implementation of mitigation strategies.

## Figures and Tables

**Table 1:** Statements used to measure outcome beliefs about controlling aggression (OB), social influence (SI), perceived possibility to control aggression (PP), total number of aggression control strategies tried (NST), perception of aggression as a problem (PERC) and willingness to control aggression (WILL).

	Item	Statement	Scale
Outcome beliefs (OB)	OB_1_	A reduction in mixing aggression would create more profit for my farm	1 = strongly disagree to 7 = strongly agree
	OB_2_	A reduction in mixing aggression would improve animal welfare on my farm	
	OB_3_	A reduction in mixing aggression would improve my job satisfaction	
Social Influence (SI)		*How much does the opinion of this person/group affect your decisions with regard to mixing aggression?*	
	SI_1_	Veterinarians	1 = not at all to 7 = very much
	SI_2_	Researchers	
	SI_3_	Wholesale/retail trade	
	SI_4_	Agricultural advisor	
	SI_5_	Slaughterhouse staff	
	SI_6_	Consumers	
	SI_7_	Other pig farmers	
	SI_8_	Quality assurance body	
	SI_9_	Levy body	
Perceived possibility (PP)	PP_1_	I am aware of methods to reduce mixing aggression	1 = strongly disagree to 7 = strongly agree
	PP_2_	I or my colleagues have the possibility to make changes in management which would reduce mixing aggression	
	PP_3_	I or my colleagues have the time available to reduce mixing aggression	
	PP_4_	I have the financial resources to reduce mixing aggression	
Perception (PERC)	PERC	Mixing aggression is a problem on my farm	1 = strongly disagree to 7 = strongly agree
Number of strategies tried (NST)	NST	*Please indicate which technique(s) you have tried to minimize aggression between growing/finishing pigs:* Purposely mix pigs from neighboring pens Odor masking agents Barriers or get-way areas e.g., adding straw bales Evening/night time mixing Distraction material/toys Pre-weaning socialization/allowing litters to mix before weaning Mixed-weight pens Mix into large groups Stresnil/azaperone (a sedative) Other (please specify)	Yes/no
Willingness (WILL)	WILL	In the near future how likely are you to implement strategies to reduce mixing aggression between growing/finishing pigs?	1 = not likely at all to 7 = very likely

**Table 2:** Mean number of pigs per farm at any moment in time and housing system for weaners, grower/finishers, lactating and dry sows.

Stage of Production	Mean Number of Pigs ± Std. (Number of Farmers to Disclose)	Range	Housing Percentage (Number)			
			Indoor	Outdoor	Combined	Undisclosed
Weaners	3252 ± 5736.8 (91)	0 *–40,008	68.9% (84)	5.7% (7)	4.9% (6)	19.7% (24)
Growers;	3625.2 ± 4382.7 (92)	0 *–23,300	74.6% (91)	4.1% (5)	3.3% (4)	18% (22)
Finishers			73.8% (90)	2.5% (3)	4.1% (5)	19.7% (24)
Lactating Sows;	959.6 ± 1722.4 (96)	0 *–12,500	60.7% (74)	14.8% (18)	3.3% (4)	20.5% (25)
Dry Sows			62.3% (76)	12.3% (15)	4.1% (5)	20.5% (25)

* Some farmers reported keeping zero pigs either because they do not keep this stage of production or because they are now retired.

**Table 3:** Percentage of respondents who had tried each aggression mitigation strategy and the median usefulness score for each strategy (from 1 = not useful at all to 7 = very useful). Different letters indicate significantly different usefulness scores at *p* < 0.05.

Aggression Mitigation Strategy	Percentage Tried (Number)	Median Usefulness Score (1–7)
Purposely mixing pigs from neighboring pens	34.4% (42)	4 ^b^
Odor masking agents	38.5% (47)	4.5 ^a,b^
Barriers or get away areas	40.2% (49)	5 ^a,b^
Evening/night time mixing	15.6% (19)	5 ^a,b^
Distraction material/toys	73.8% (90)	5 ^a,b^
Pre-weaning socialization	38.5% (47)	6 ^a^
Mixed-weight pens	21.3% (26)	4 ^b^
Mix into large groups	45.1% (55)	5 ^a,b^
Stresnil/azaperone (sedative)	13.1% (17)	5 ^a,b^

**Table 4:** Reliability of the standardized confirmatory factor analysis.

Construct	Indicators	Standardized Loadings (Std Error)	Composite Reliability (AVE)	Measurement Model
Outcome beliefs about controlling aggression (OB)	Cronbach’s *a*	0.93	0.93 (0.813)	*χ*^2^ = 85.24 df = 53 *χ*^2^/df = 1.61 RMSEA = 0.071 SRMR = 0.053 CFI = 0.962 SC = 0.027
	PO_1_	0.88 (0.03)		
	PO_2_	0.96 (0.02)		
	PO_3_	0.86 (0.04)		
Social Influence (SI)	Cronbach’s *a*	0.90	0.90 (0.512)	
	SI_1_	0.41 (0.08)		
	SI_2_	0.71 (0.06)		
	SI_3_	0.76 (0.06)		
	SI_4_	0.80 (0.05)		
	SI_5_	0.69 (0.06)		
	SI_6_	0.80 (0.04)		
	SI_7_	0.67 (0.07)		
	SI_8_	0.76 (0.05)		
	SI_9_	0.74 (0.06)		

## Supplementary Materials

The following are available online at https://osf.io/7cegq/?view_only=0bbed97785e447a6bcb9c24df3549abf, Survey, Data.

## Appendix A

**Table A1:** Spearman correlation matrix among variables used in the structural model whereby * *p* < 0.05, ** *p* < 0.01.

	OB_1_	OB_2_	OB_3_	SI_1_	SI_2_	SI_3_	SI_4_	SI_5_	SI_6_	SI_7_	SI_8_	SI_9_	PP_1_	PP_3_	PP_3_	PP_4_	NST	PERC	WILL
**OB_1_**																			
**OB_2_**	0.853 **																		
**OB_3_**	0.746 **	0.813 **																	
**SI_1_**	0.075	0.104	0.121																
**SI_2_**	0.214 *	0.233 **	0.256 **	0.262 **															
**SI_3_**	0.092	0.101	0.099	0.239 **	0.601 **														
**SI_4_**	0.155	0.116	0.161	0.304 **	0.604 **	0.559 **													
**SI_5_**	0.026	0.046	0.160	0.259 **	0.466 **	0.574 **	0.516 **												
**SI_6_**	−0.008	0.003	0.062	0.214 *	0.521 **	0.684 **	0.580 **	0.602 **											
**SI_7_**	0.155	0.074	0.173	0.318 **	0.430 **	0.405 **	0.653 **	0.433 **	0.430 **										
**SI_8_**	0.016	0.040	0.122	0.289 **	0.527 **	0.513 **	0.645 **	0.496 **	0.662 **	0.477 **									
**SI_9_**	0.030	0.048	0.056	0.326 **	0.445 **	0.571 **	0.555 **	0.542 **	0.685 **	0.523 *	0.551 **								
**PP_1_**	0.264 **	0.277 **	0.239 **	0.144	−0.134	−0.121	−0.045	−0.078	−0.210	−0.051	−0.066	−0.009							
**PP_2_**	0.190 *	0.275	0.349 *	0.069	0.181 *	0.171	0.275 **	0.230 *	0.263 **	0.156	0.295 **	0.196 *	0.230 *						
**PP_3_**	−0.026	0.18	0.103	0.114	0.029	0.049	0.159	0.096	0.170	0.050	0.210 *	0.079	0.209 *	0.566 **					
**PP_4_**	0.017	0.000	0.107	0.051	−0.030	0.023	0.032	0.080	0.048	−0.002	0.093	0.128	0.288 **	0.195	0.445 **				
**NST**	−0.046	−0.028	0.146	0.085	0.119	0.176	0.116	0.249 **	0.154	0.192 *	0.170	0.089	0.125	0.255 **	0.165	0.095			
**PERC**	0.317 **	0.285 **	0.300 **	−0.033	0.213 *	0.069	0.042	0.103	0.111	0.015	0.094	0.046	−0.142	0.059	−0.199 *	0.042	0.092		
**WILL**	0.295 **	0.373 **	0.422 **	0.054	0.189 *	0.049	0.161	0.141	0.080	0.239 **	0.143	0.094	0.275 **	0.363 **	0.145	−0.007	0.152	0.125	
Median	5	5	5	6	5	3	4	2	3	5	4	3	6	4.5	4	4	3	3	4
